# Comparison of thromboelastometry with procalcitonin, interleukin 6, and C-reactive protein as diagnostic tests for severe sepsis in critically ill adults

**DOI:** 10.1186/cc9284

**Published:** 2010-10-07

**Authors:** Michael Adamzik, Martin Eggmann, Ulrich H Frey, Klaus Görlinger, Martina Bröcker-Preuß, Günter Marggraf, Fuat Saner, Holger Eggebrecht, Jürgen Peters, Matthias Hartmann

**Affiliations:** 1 Klinik für Anästhesiologie und Intensivmedizin, Universitätsklinikum Essen und Universität Duisburg-Essen, Hufelandstr. 55, 45130 Essen, Germany; 2 Klinik für Endokrinologie, Zentrallabor Bereich Forschung und Lehre, Universitätsklinikum Essen, Hufelandstr. 55, 45122 Essen, Germany; 3 Klinik für Thorax- und kardiovaskuläre Chirurgie, Universitätsklinikum und Universität Duisburg-Essen, Hufelandstr. 55, 45122 Essen, Germany; 4 Klinik für Allgemein- und Transplantationschirurgie, Universitätsklinikum Essen und Universität Duisburg-Essen, Hufelandstr. 55, 45122 Essen, Germany; 5 Klinik für Kardiologie, Universitätsklinikum Essen und Universität Duisburg-Essen, Hufelandstr. 55, 45122 Essen, Germany

## Abstract

**Introduction:**

Established biomarkers for the diagnosis of sepsis are procalcitonin, interleukin 6, and C-reactive protein. Although sepsis evokes changes of coagulation and fibrinolysis, it is unknown whether thromboelastometry can detect these alterations. We investigated whether thromboelastometry variables are suitable as biomarkers for severe sepsis in critically ill adults.

**Methods:**

In the observational cohort study, blood samples were obtained from patients on the day of diagnosis of severe sepsis (*n *= 56) and from postoperative patients (*n *= 52), and clotting time, clot formation time, maximum clot firmness, alpha angle, and lysis index were measured with thromboelastometry. In addition, procalcitonin, interleukin 6, and C-reactive protein levels were determined. For comparison of biomarkers, receiver operating characteristic (ROC) curves were used, and the optimal cut-offs and odds ratios were calculated.

**Results:**

In comparison with postoperative controls, patients with sepsis showed an increase in lysis index (97% ± 0.3 versus 92 ± 0.5; *P *< 0.001; mean and SEM) and procalcitonin (2.5 ng/ml ± 0.5 versus 30.6 ± 8.7; *P *< 0.001). Clot-formation time, alpha angle, maximum clot firmness, as well as interleukin 6 and C-reactive protein concentrations were not different between groups; clotting time was slightly prolonged. ROC analysis demonstrated an area under the curve (AUC) of 0.901 (CI 0.838 - 0.964) for the lysis index, and 0.756 (CI 0.666 - 0.846) for procalcitonin. The calculated cut-off for the lysis index was > 96.5%, resulting in a sensitivity of 84.2%, and a specificity of 94.2%, with an odds ratio of 85.3 (CI 21.7 - 334.5).

**Conclusions:**

The thromboelastometry lysis index proved to be a more reliable biomarker of severe sepsis in critically ill adults than were procalcitonin, interleukin 6, and C-reactive protein. The results also demonstrate that early involvement of the hemostatic system is a common event in severe sepsis.

## Introduction

Sepsis is a common cause of death in critically ill patients, and early diagnosis is mandatory to improve the prognosis. Commonly used biomarkers like procalcitonin, C-reactive protein, and interleukin 6 are produced by the host in response to infections. However, the concentrations of these biomarkers can increase in patients with trauma or surgery, even without infection, and, therefore, their diagnostic value in critically ill patients is far from perfect [1].

In patients with sepsis, activation of hemostasis is of marked pathophysiologic relevance, as it is associated with increased mortality [2]. As the mechanism, fibrin deposition in the vasculature, leading to ischemia and multiorgan failure, is assumed [3]. Only sparse information, however, is available on the use of thromboelastometry in sepsis. This method measures the mechanical properties of a forming clot in whole-blood samples in a time-dependent fashion and is an increasingly accepted point-of-care method for monitoring and therapy of hemostatic disturbances [4]. In a recent study, we demonstrated that endotoxinemia can be detected with thromboelastometry under *in vitro *conditions [5]. Thromboelastometric variables remained within reference ranges during the course of critically illness in 30 patients with sepsis [6]. In another study, however, early changes in thromboelastometry values were demonstrated in endotoxin-treated pigs [7].

The aim of the present study was to investigate the value of thromboelastometry variables as potential biomarkers of sepsis in critically ill adults and to compare these hemostasis-related biomarkers with the established markers procalcitonin, interleukin 6, and C-reactive protein.

## Materials and methods

### Patients

The study was reviewed and approved by the Ethics Committee of the University Hospital Essen. In detail, informed written consent was given by both postoperative patients and probands. Informed consent of patients with sepsis was waived by the ethics committee, but written informed consent for the use of data was acquired by the surviving patients after recovery from the disease. Over a period of 2 years, 56 patients admitted to an ICU of the University Hospital of Essen were considered eligible for the study if they fulfilled the criteria for severe sepsis (sepsis group) [8]. As the second group, patients admitted to the ICU after surgery but without the criterion of sepsis were chosen (postoperative group). Groups were not matched. A detailed characterization of patients and controls is given in Table 1. As a third group, healthy probands were chosen (probands group). In all groups, whole-blood samples were subjected to thromboelastometry (ROTEM 05; Pentapharm, Germany). Samples from septic and postoperative patients were drawn within 24 hours of diagnosis and surgery, respectively. Furthermore, procalcitonin, interleukin 6, and C-reactive protein concentrations as well as SAPS II and SOFA scores were determined in these groups at the same time [9,10].

**Table 1:** Characteristics of patients with sepsis and postoperative patients

Patient characteristics	Sepsis	Postoperative patients
Number of patients	56	52
Age, years	54 ± 17	55 ± 17
Male/female	31/25	28/24
Weight, kg	79.9 ± 23.5	74.9 ± 26.5

Primary diagnosis		

Gastrointestinal cancer	7	18
Gastrointestinal disease	16	8
Cancer, other	5	12
Urogenital cancer	3	9
	11	0
Lung disease	9	0
Urogenital disease	3	1
Other diseases	1	4
Lung cancer	1	0

Disease severity		

CVVHD	33	0
Mechanical ventilation, %	100	100
SAPS II score	51.4 ± 14.9	20.8 ± 9.0
SOFA score	12.5 ± 3.9	3.85 ± 2.6

Infection type		

Gram-positive isolates, %	28	0
Gram-negative isolates, %	49	0
Viral isolates, %	0	0
Fungal isolates, %	11	0

Included are biometric data, primary diagnosis, disease severity, and infection type as diagnosed within 24 hours after admission. Data are given as mean and standard error of the mean. CVVHD, continuous venovenous hemodiafiltration.

### Thromboelastometry

Whole-blood coagulation properties of citrated blood samples were determined by using thromboelastometry. To exclude potential effects of heparin on coagulation, 20 μl heparinase was added to the samples according to the manufacturer's recommendations (Pentapharm, München, Germany). Thereafter, samples were subjected to thromboelastometry (ROTEM 05; Pentapharm), and coagulation was initiated by addition of CaCl_2 _(20 μl, 0.2 *M *CaCl_2_, NaTEM test). Clotting time (CT), clot-formation time (CFT), maximum clot firmness (MCF), alpha angle, and the 60-minute lysis index were determined.

### Assays for procalcitonin, interleukin 6, and C-reactive protein concentrations

For the determination of procalcitonin concentration, the Liaison Brahms PCT assay (Diasorin S.p.A., Sallugia, Italy) was used. C-reactive protein was measured by using the CRP wide-range assay of the Avidia 1650 chemistry system (Bayer Healthcare LLC, Leverkusen, Germany). Interleukin 6 was determined by using an Immulite 2000 systems analyzer and reagents (Siemens Healthcare Diagnostics Products Ltd., Duisburg, Germany).

### Statistical analysis

Values for the thromboelastometry variables and concentrations of procalcitonin, interleukin 6, and C-reactive protein in patients with and without severe sepsis are given as mean and standard error of the mean (SEM), as well as median and 25^th ^and 75^th ^percentiles. The Shapiro-Wilk test excluded a normal distribution for several values. Therefore, the Mann-Whitney test was used for statistical evaluation. For the comparison of biomarkers, receiver operating characteristic (ROC) curves were used, and these results are given as area under the curve (AUC), 95% confidence interval (CI), and asymptotic significance (*P *value). Furthermore, the optimal cut-off value for each biomarker was calculated, and the corresponding sensitivities and specifities are presented. Optimal sensitivity and specificity were defined as those yielding the minimal value for (1 - sensitivity)^2 ^+ (1 - specificity)^2^, as described [11]. With the calculated optimal cut-off values, the odds ratios were calculated along with the respective 95% CIs, as well as the significance values, by using the χ^2 ^test. SPSS Version 16 (SPSS Inc., Chicago, IL, USA) was used for all statistical procedures, and an *a priori *alpha error *P *of < 0.05 was considered statistically significant.

## Results

### Thromboelastometry variables in probands and postoperative patients

In comparison with probands, postoperative patients showed an increased hemostasis potential. Thromboelastometry variables were characterized by a shorter clotting time and clot-formation time, as well as increased alpha angle and maximum clot firmness. Remarkably, the lysis index was not different in probands and postoperative patients (Table 2).

**Table 2:** Thromboelastometry values in patients with sepsis, postoperative patients, and probands

Thromboelastometry	Sepsis Mean [SEM] Median [quartiles]	Postoperative patients Mean [SEM] Median [quartiles]	Probands Mean [SEM] Median [quartiles]	Mann-Whitney test Sepsis vs. postop, Sepsis vs. probands Postop vs. probands
**Lysis index** %	97.0 ± 0.3 98.0[97.3-98.0]	92.0 ± 0.5 92.0 [90.0-95.0]	92.6 ± 0.7 93.0 [91.3-95.0]	< 0.001; < 0.001; 0.53
**Clotting time** Seconds	546 ± 30 513 [406-639]	434 ± 16 453 [386-485]	765 ± 33 774 [668-865]	0.012; < 0.001; < 0.001
**Alpha-angle** Degree	55.4 ± 1.5 56.0[ 48.0-65.0]	59.3 ± 1.5 62.0 [56.0-67.0]	48.4 ± 1.8 46.5 [43.2-54.0]	0.085, 0.003; < 0.001
**Clot-formation time** Seconds	229 ± 19 187 [136-271]	193 ± 17 166 [122-196]	259 ± 17 262 [206-303]	0.095; 0.01; < 0.001
**Max. clot firmness** mm	55.4 ± 1.5 54.5 [49.3-65.0]	55.8 ± 1.3 57.0 [50.0-62.0]	51.8 ± 1.0 52.0 [47.5-54.8]	0.858; 0.10; 0.032

Values are given as mean and standard error of the mean as well as median and 25^th ^and 75^th ^percentiles (quartiles). The Mann-Whitney test was used for statistical evaluation.

### Thromboelastometry variables in critically ill patients with and without severe sepsis

In comparison with postoperative patients, sepsis patients showed an increased lysis index (97.0% ± 0.3 versus 92.0 ± 0.5; *P *< 0.001) Clot-formation time, alpha angle, and maximum clot firmness were not significantly different between groups (Table 2), but the clotting time was slightly prolonged.

### Conventional biomarkers in critically ill patients with and without severe sepsis

Procalcitonin, interleukin 6, and C-reactive protein concentrations were tested for differences between patients with and without sepsis. Procalcitonin concentration averaged 2.5 ng/ml ± 0.5 in postoperative patients but 30.6 ng/ml ± 8.7 in patients with severe sepsis (*P *< 0.001). Neither interleukin 6 nor C-reactive protein concentrations were significantly different between patients with and without sepsis (Table 3). In both postoperative and sepsis patients, mean values for procalcitonin, interleukin 6, and C-reactive protein exceeded the reference interval by far (Table 3).

**Table 3:** Conventional biomarkers of sepsis in patients with sepsis and postoperative patients

Biomarker	Sepsis Mean [SEM] Median [quartiles]	Postoperative patients Mean [SEM] Median [quartiles]	Reference values	Mann-Whitney test Sepsis vs. postop *P *value
**Procalcitonin** ng/ml	**30.6 ± 8.7** **5.5 [1.5-24.3]**	**2.5 ± 0.5** **1.4 [0.4-3.3]**	** < 0.5 (probands)**	** < 0.001**
**Interleukin 6** pg/ml	**1,054 ± 426** **114 [36-592]**	**313 ± 40** **188 [120-422]**	**0-3.4**	**0.108**
**C-reactive protein** mg/dl	**14.7 ± 1.3** **13.6 [6.1-21.8]**	**12.5 ± 0.7** **12.1 [8.9-16.2]**	**0-0.5**	**0.563**

Reference values of the biomarker assays are given. Values are given as mean and standard error of the mean as well as median and 25^th ^and 75^th ^percentiles (quartiles). The Mann-Whitney test was used for statistical evaluation.

### Comparison of thromboelastometry variables and conventional biomarkers for the diagnosis of severe sepsis in critical ill adults

**Figure 1:**
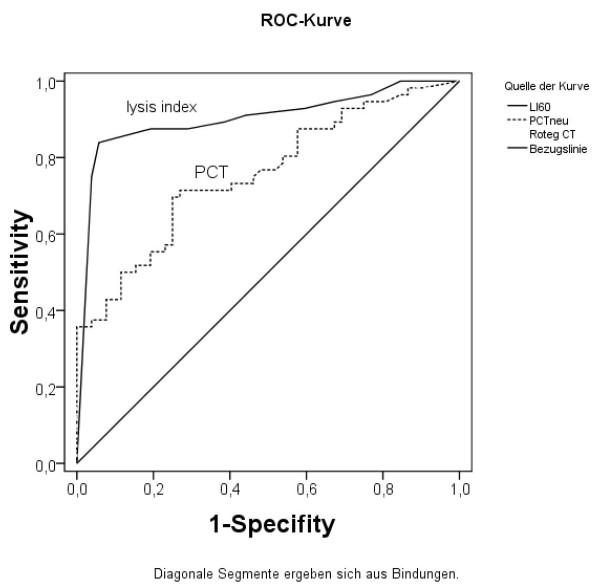
**Receiver operating characteristic curves comparing thromboelastometry lysis index and procalcitonin concentration for the diagnosis of severe sepsis in critically ill patients**.

As shown above, thromboelastometry lysis index and procalcitonin concentration were different in postoperative and sepsis patients. To further investigate the diagnostic value of these variables as potential biomarkers of severe sepsis in critical illness, a ROC curve analysis was performed. Furthermore, the 95% confidence intervals (CI), as well as the asymptotic significance niveaus were determined. The best accuracy was yielded by the lysis index, with an AUC of 0.901 (CI 0.838 - 0.964; *P *< 0.001), followed by procalcitonin concentration (AUC 0.75; CI 0.666 - 0.846; *P *< 0.001). The ROC curves for these variables are shown in Figure 1. Comparison of the lysis index in probands and patients with sepsis, respectively, demonstrated that the variable was capable of detecting differences between these groups with high accuracy, too (AUC 0.890; CI 0.845 - 0.977; *P *< 0.001).

### Optimal cut-off values for lysis index and procalcitonin concentration for the diagnosis of sepsis in critically ill adults

Optimal cut-off values were determined as described in the Methods section. For the lysis index, the optimum cut-off was > 96.5%, resulting in a sensitivity of 84.2% and a specificity of 94.2%. Applying this cut-off for the comparison of probands and sepsis patients, respectively, resulted in a sensitivity of 83.9% and a specificity of > 99%. The optimum cut-off for procalcitonin concentration was > 2.58 ng/ml, resulting in a sensitivity of 70.2% and a specificity of 75.0%.

### Odds ratios for the biomarkers for the diagnosis of sepsis in critically ill patients

When applying these calculated optimum cut-off values for the biomarkers, the resulting odds ratios for the detection of severe sepsis in critically ill patients were 85.3 (CI 21.7 - 334.5; *P *< 0.001) for the lysis index and 6.3 (CI 2.7 - 14.4; *P *< 0.001) for procalcitonin concentration.

## Discussion

Our results demonstrate that the thromboelastometry lysis index can discriminate intensive care patients with severe sepsis from postoperative patients and probands. Furthermore, comparison of thromboelastometry findings with the conventional biomarkers procalcitonin, interleukin 6, and C-reactive protein demonstrates a superior accuracy of the thromboelastometry lysis index in identifying patients with severe sepsis in critically ill patients. Finally, the data indicate that the fibrinolytic system is inhibited in nearly all patients with severe sepsis.

Thromboelastometry is a point-of-care method capable of determining the kinetics of clot formation and clot lysis in whole-blood samples, thereby assessing the viscoelastic properties of the clot [12]. The clotting variables obtained by thromboelastometry include the clotting time, representing the time to onset of coagulation, the clot-formation time and alpha angle, both of which describe the kinetics of clot formation, and the maximum clot firmness, describing the mechanical properties of the clot, which depends on both platelet count and fibrin polymerization. For the determination of the lysis index, the clot firmness prevailing 60 minutes after maximum clot firmness is reached, is divided by the maximum clot firmness. Thromboelastometry is widely accepted in cardiac and liver transplantation surgery [13,14], but studies on its use in sepsis are sparse. Although recent data obtained in experimental sepsis and small patient cohorts suggest that sepsis-induced alterations in hemostasis might be detected with thromboelastometry, it is unknown whether thromboelastometry variables might serve as biomarkers for the diagnosis of severe sepsis [5-7,15].

**Figure 2:**
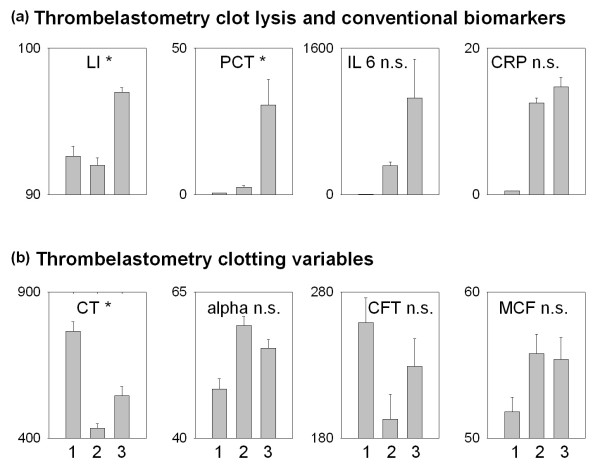
**Thromboelastometry variables and conventional biomarkers in probands (1), postoperative patients (2), and patients with sepsis (3), respectively**. Data are given as mean and standard error of the mean. The asterisks denote significant differences between postoperative and sepsis patients.

In Figure 2, the results obtained by both the thromboelastometry lysis index and conventional biomarkers (a) as well as the thromboelastometry clotting variables (b) are summarized. The figure demonstrates that the lysis index is not different in probands and postoperative patients but is significantly higher in patients with severe sepsis. Similarly, procalcitonin slightly increases in postoperative patients and shows a further marked increase in patients with severe sepsis. In contrast, interleukin 6 and C-reactive protein are markedly higher in postoperative patients, but no further increase is found in patients with severe sepsis. Thus, thromboelastometry lysis index and procalcitonin, but not interleukin 6 and C-reactive protein, are capable of detecting patients with severe sepsis in critically ill adults. Concerning the thromboelastometry clotting variables clotting time, alpha angle, and clot-formation time, the present study demonstrates an increased hemostasis potential in postoperative patients (Figure 2b); maximum clot firmness was not different in these groups. The differences observed between postoperative patients and patients with severe sepsis were small, not significant in most cases, and thus do not allow detection of the patients with severe sepsis.

It is a main result of the present study that the thromboelastometry lysis index was increased in patients with severe sepsis in comparison with probands and postoperative patients, suggesting that the function of the fibrinolytic system is markedly inhibited. Whereas clot firmness decreased by 8% after 1 hour in patients without sepsis and in probands, clot firmness decreased by only 3% in patients with severe sepsis. The fact that the thromboelastometry lysis index was the most reliable biomarker tested for the diagnosis of severe sepsis in critically ill patients in our study demonstrates that thromboelastometry is capable of detecting changes in the fibrinolytic system in severe sepsis. Furthermore, because changes in thromboelastometry variables were seen on the day of diagnosis of severe sepsis, our data demonstrate an early involvement of the fibrinolysis system occurring in almost all patients (84.2%) with severe sepsis. In this regard, it is important that the thromboelastometry lysis index is not different in probands and postoperative patients. Thus, an inhibition of fibrinolysis was found to be an integral part of the host response to severe infection but not to surgery.

Several reports address fibrinolysis in sepsis as well as the potential mechanisms involved [16]. Boudjeltia *et al. *[17] demonstrated a decrease in plasma fibrinolysis in sepsis, which was associated with organ dysfunction. As a mechanism, an increase in plasminogen activator inhibitor 1 (PAI-1), which is produced by endothelium and liver, has been demonstrated [18]. As activated protein C degrades PAI-1 and inhibits thrombin activable fibrinolysis inhibitor (TAFI), the decreased concentrations in activated protein C in sepsis may contribute to the inhibition of fibrinolysis in sepsis [19-21]. The importance of the fibrinolytic system in sepsis also has been demonstrated in genetically modified mice, showing that endotoxin-induced fibrin deposition in organs of mice deficient for tPA or uPA was more extensive than that in wild-type mice, and the opposite held true for PAI-1-deficient mice [22]. Although the latter work suggests a deleterious effect of the reduced fibrinolytic rate in an endotoxin model of sepsis, others describe that local thrombosis/fibrin-deposition limits the survival and dissemination of microbial pathogens in mice [23]. Thus, reduced fibrinolysis in sepsis probably reduces the invasion by and the spreading of bacteria but favors disseminated intravascular coagulation, leading to organ ischemia and multiorgan failure.

The present study has limitations. The number of patients in the cohort was limited, and the sensitivity and specifity of thromboelastometry values and of conventional biomarkers for the diagnosis of sepsis might differ in other cohorts and require further studies. Furthermore, the clinical use of thromboelastometry variables as a biomarker for severe sepsis might be limited by the fact that citrated whole-blood samples have to be processed within a short time frame, and that the method is time consuming when compared with automated laboratory methods. It is a fact that the groups in the present study were heterogeneous. However, we compared several biomarker and the best biomarker, the lysis index, showed an exceedingly high odds ratio of 85.3.

## Conclusions

The results of the present study demonstrate that severe sepsis is associated with reduced fibrinolysis, as evidenced by thromboelastometry. The lysis index proved to be a better biomarker for sepsis in critical illness than procalcitonin, interleukin 6, or C-reactive protein. The fact that an inhibition of fibrinolysis occurred in nearly all patients with severe sepsis but not in postoperative patients suggests an important role of the fibrinolytic system in the pathophysiology of severe sepsis.

## Key messages

• In comparison with probands and postoperative patients, the thromboelastometry lysis index is markedly increased in patients with severe sepsis.

• The thromboelastometry lysis indexed proved to be the best biomarker of sepsis in critically ill adults, followed by procalcitonin. Interleukin 6 and C-reactive protein were not different.

• The fact that clot lysis is reduced in almost all patients with severe sepsis suggests an important role of the fibrinolytic system in severe sepsis.

## Abbreviations

AUC: area under curve; CFT: clot-formation time; CI: confidence interval; CRP: C-reactive protein; CT: clotting time; MCF: maximum clot firmness; ROC curve: receiver operating characteristic curve.

## Competing interests

The authors declare that they have no competing interests.

## Authors' contributions

Conception of the study was done by MH. MA, ME, GM, FS, HE, and MH contributed to data acquisition. ME, KG, and MH measured thromboelastometry variables. MB measured the conventional sepsis marker. Data were analyzed by MH, MA, UF, and JP. Drafting of the manuscript was done by MH, MA, and JP. All authors critically revised and approved the manuscript.

## References

[B1] Uzzan B Cohen R Nicolas P Cucherat M Perret GY Procalcitonin as a diagnostic test for sepsis in critically ill adults and after surgery or trauma: a systematic review and meta-analysis Crit Care Med 2006 34 1996 2003 10.1097/01.CCM.0000226413.54364.36 16715031

[B2] Bakhtiari K Meijers JC de Jonge E Levi M Prospective validation of the International Society of Thrombosis and Haemostasis scoring system for disseminated intravascular coagulation Crit Care Med 2004 32 2416 2421 10.1097/01.CCM.0000147769.07699.E3 15599145

[B3] Gando S Microvascular thrombosis and multiple organ dysfunction syndrome Crit Care Med 2010 38 S35 S42 10.1097/CCM.0b013e3181c9e31d 20083912

[B4] Ganter MT Hofer CK Coagulation monitoring: current techniques and clinical use of viscoelastic point-of-care coagulation devices Anesth Analg 2008 106 1366 1375 10.1213/ane.0b013e318168b367 18420846

[B5] Zacharowski K Sucker C Zacharowski P Hartmann M Thrombelastography for the monitoring of lipopolysaccharide induced activation of coagulation Thromb Haemost 2006 95 557 561 16525587 10.1160/TH05-06-0420

[B6] Velik-Salchner C Streif W Innerhofer P Maier S Knotzer H Pajk W Klingler A Mittermayr M Haas T Endotoxinemia-induced changes in coagulation as measured by rotation thrombelastometry technique and conventional laboratory tests: results of a pilot study on pigs Blood Coagul Fibrinolysis 2009 20 41 46 10.1097/MBC.0b013e32831be9ad 19129727

[B7] Daudel F Kessler U Folly H Lienert JS Takala J Jakob SM: Thromboelastometry for the assessment of coagulation abnormalities in early and established adult sepsis: a prospective cohort study Crit Care 2009 13 R42 10.1186/cc7765 19331653 PMC2689486

[B8] Levy MM Fink MP Marshall JC Abraham E Angus D Cook D Cohen J Opal SM Vincent JL Ramsay G SCCM/ESICM/ACCP/ATS/SIS 2001 SCCM/ESICM/ACCP/ATS/SIS International Sepsis Definitions Conference Crit Care Med 2003 31 1250 2001 10.1097/01.CCM.0000050454.01978.3B 12682500

[B9] Le G Jr Lemeshow S Saulnier F A new Simplified Acute Physiology Score (SAPS II) based on a European/North American multicenter study JAMA 1993 270 2957 2963 10.1001/jama.270.24.2957 8254858

[B10] Ferreira FL Bota DP Bross A Mélot C Vincent JL Serial evaluation of the SOFA score to predict outcome in critically ill patients JAMA 2001 286 1754 1758 10.1001/jama.286.14.1754 11594901

[B11] Akobeng AK Understanding diagnostic tests 3: receiver operating characteristic curves Acta Paediatr 2007 96 644 647 10.1111/j.1651-2227.2006.00178.x 17376185

[B12] Ebinger T Ruland A Lakner M Schwaiger M Validity, regulatory registration and approval of ROTEM thromboelastometry Blood Coagul Fibrinolysis 2010 21 106 107 10.1097/MBC.0b013e3283306e28 20065662

[B13] Gillies BS Thromboelastography and liver transplantation Semin Thromb Hemost 1995 21 Suppl 4 45 49 8747687

[B14] Dunning J Versteegh M Fabbri A Pavie A Kolh P Lockowandt U Nashef SA EACTS Audit and Guidelines Committee Guideline on antiplatelet and anticoagulation management in cardiac surgery Eur J Cardiothorac Surg 2008 34 73 92 10.1016/j.ejcts.2008.02.024 18375137

[B15] Gonano C Sitzwohl C Meitner E Weinstabl C Kettner SC Four-day antithrombin therapy does not seem to attenuate hypercoagulability in patients suffering from sepsis Crit Care 2006 10 R160 10.1186/cc5098 17107615 PMC1794466

[B16] Levi M van der Poll T Inflammation and coagulation Crit Care Med 2010 38 S26 S34 10.1097/CCM.0b013e3181c98d21 20083910

[B17] Boudjeltia KZ Ollieuz S Piagnerelli M Biston P Cauchie P Vincent JL Brohee D Vanhaeverbeek M Plasma fibrinolysis is related to the degree of organ dysfunction but not to the concentration of von Willebrand factor in critically ill patients Thromb J 2009 7 10 19538758 10.1186/1477-9560-7-10 PMC2711920

[B18] van der Poll T Levi M Büller HR van Deventer SJ de Boer JP Hack CE ten Cate JW Fibrinolytic response to tumor necrosis factor in healthy subjects J Exp Med 1991 174 729 732 10.1084/jem.174.3.729 1714936 PMC2118940

[B19] Neyrinck AP Liu KD Howard JP Matthay MA Protective mechanisms of activated protein C in severe inflammatory disorders Br J Pharmacol 2009 158 1034 1047 10.1111/j.1476-5381.2009.00251.x 19466992 PMC2785525

[B20] Mosnier LO Meijers JC Bouma BN Regulation of fibrinolysis in plasma by TAFI and protein C is dependent on the concentration of thrombomodulin Thromb Haemost 2001 85 5 11 11204587

[B21] Savioli M Cugno M Polli F Taccone P Bellani G Spanu P Pesenti A Iapichino G Gattinoni L Tight glycemic control may favor fibrinolysis in patients with sepsis Crit Care Med 2009 37 424 431 10.1097/CCM.0b013e31819542da 19114908

[B22] Carmeliet P Schoonjans L Kieckens L Ream B Degen J Bronson R De Vos R van den Oord JJ Collen D Mulligan RC Physiological consequences of loss of plasminogen-activator gene-function in mice Nature 1994 368 419 424 10.1038/368419a0 8133887

[B23] Sun H Wang X Degen JL Ginsburg D Reduced thrombin generation increases host susceptibility to group A streptococcal infection Blood 2009 113 1358 1364 10.1182/blood-2008-07-170506 19056689 PMC2637198

